# Results after arthroscopic treatment of iliopsoas impingement after total hip arthroplasty

**DOI:** 10.1007/s00402-020-03623-z

**Published:** 2020-10-12

**Authors:** A. Zimmerer, M. Hauschild, R. Nietschke, M. M. Schneider, G. Wassilew, C. Sobau, W. Miehlke

**Affiliations:** 1 ARCUS Sportklinik, Rastatterstr. 17-19, 75179 Pforzheim, Germany; 2 Department of Orthopedics and Orthopedic Surgery, University Medicine Greifswald, Ferdinand-Sauerbruch-Straße, 17475 Greifswald, Germany; 3 University of Witten/Herdecke, Alfred-Herrhausen-Straße 50, 58455 Witten, Germany

**Keywords:** Total hip replacement, Iliopsoas tendon, Revision hip surgery, Complication, Outcome, Hip prosthesis

## Abstract

**Background:**

A cause of groin pain after total hip arthroplasty (THA) is mechanical irritation or impingement of the iliopsoas tendon. The incidence is about 4%. If conservative therapy fails, an arthroscopic release of the iliopsoas tendon can be performed. The aim of the study was to assess the mid-term clinical outcome after arthroscopic release. We hypothesize that good results can be achieved by a minimally invasive endoscopic procedure.

**Methods:**

Using our in-house database, all patients who received an endoscopic release of the iliopsoas tendon due to mechanical irritation after THA were identified. Inclusion criteria were mechanical irritation of the iliopsoas tendon after cementless THA with minimal acetabular component prominence. Exclusion criteria were marked prominence of the acetabular component and groin pain after THA for any other reason. In these patients, the modified Harris Hip Score (mHHS), the pain level using the numerical analogue scale and the UCLA Activity Score were measured. The mean follow-up period was 7 ± 3.8 (2.6–11.7) years.

**Results:**

25 patients were identified in whom an arthroscopic release of the iliopsoas tendon had been performed since 2007. The data of 20 patients were available at follow-up. The gender ratio was 1:1, the average age at the time of arthroscopy was 59 ± 27.7 (52–78) years. The average interval between THA and arthroscopy was 6.3 ± 4.0 (1.7–15) years. The mHHS showed a significant improvement from preoperative 31.2 ± 9.8 (17.6–47.3) to 82.0 ± 9.8 (46.2–100) points (*p* = 0.001). The pain level on the NAS decreased significantly from 8.5 ± 1.2 (7–10) to 2.5 ± 1.8 (0–6) points (*p* = 0.001). The activity level based on the UCLA Activity Score raised from 4.0 ± 2.7 (0–7) to 6.5 ± 1.8 (3–9) (*p* = 0.09).

**Conclusion:**

Mechanical irritation and impingement of the iliopsoas tendon is an important diagnosis to be considered in persistent groin pain after total hip arthroplasty. In failure of non-operative treatment, good clinical results can be achieved with arthroscopic release and the pain level can be significantly reduced.

**Level of evidence:**

IV.

## Background

The majority of patients after total hip arthroplasty (THA) are very satisfied with the result achieved [1]. However, there are patients who report pain in the groin area after THA. The cause can be very versatile: loosening of components, heterotopic ossifications, neurologic or vascular pathologies [2, 3]. Another cause that has been increasingly taken into account in recent years is the iliopsoas impingement that has been first described by Postel [4]. The incidence is estimated between 0.4 and 8.3% [5–8]. This impingement may be caused by an incorrectly positioned cup component, remaining cement or extra-long screws [9–11].

Typically, these patients complain of pain in the groin area [9, 12]. Active flexion of the hip can be painful and snapping phenomena can occur with hip flexion e.g. while stair climbing. The clinical examination often shows groin pain with resisted hip flexion or stretching of the iliopsoas tendon [13]. The diagnosis is based on clinical examination and radiographs including an anteroposterior pelvic and cross-lateral table view and an additionally computed tomography, which may show a prominence of the cup over the anterior aspect of the acetabular rim [9, 13, 14]. In addition, a magnetic resonance imaging (MRI) especially MARS-MRI or ultrasonography can be performed to demonstrate iliopsoas tendinitis [15–18].

First and foremost, conservative therapy as the gold standard should be attempted. This includes physical therapy, peritendinous injections or non-steroidal anti-inflammatory drugs (NSAIDs) [10, 19, 20]. If conservative therapy fails, surgery usually offers a relief in pain and symptoms. Several possibilities are described: acetabular component revision and open or arthroscopic/endoscopic debridement or tenotomy of the iliopsoas tendon [10, 11, 13, 21–23]. According to Chalmers et al., in patients with minimal acetabular component prominence (< 8 mm) release of the iliopsoas tendon should be preferred [24]. Various techniques are described for this procedure: an endoscopic tenotomy at the lesser trochanter, approaching the iliopsoas tendon at its distal insertion described by Ilizaliturri in 2005 and Williams in 2018 [25, 26]. Or a transcapsular release at the psoas notch described by Wettstein in 2006 [27].

Regarding the outcome, most studies report a short follow-up period averaging 8 months to 3 years [7, 10, 22, 24, 28]. To our knowledge, only one study reports outcomes with a follow-up period of more than 6 years [29]. Therefore, the aim of this study was to analyze the mid-term clinical outcome after arthroscopic release of the iliopsoas tendon.

## Methods

This is a single-center retrospective cohort study. After institutional review board approval (Ethikkommission Landesärztekammer Baden-Württemberg: F-2019-006), we identified patients via our institutional database in July 2019 and performed a retrospective analysis of prospectively collected data. We included patients that had undergone primary cementless total hip arthroplasty and subsequently sustained iliopsoas impingement. All surgeries were performed by the senior author.

**Fig. 1:**
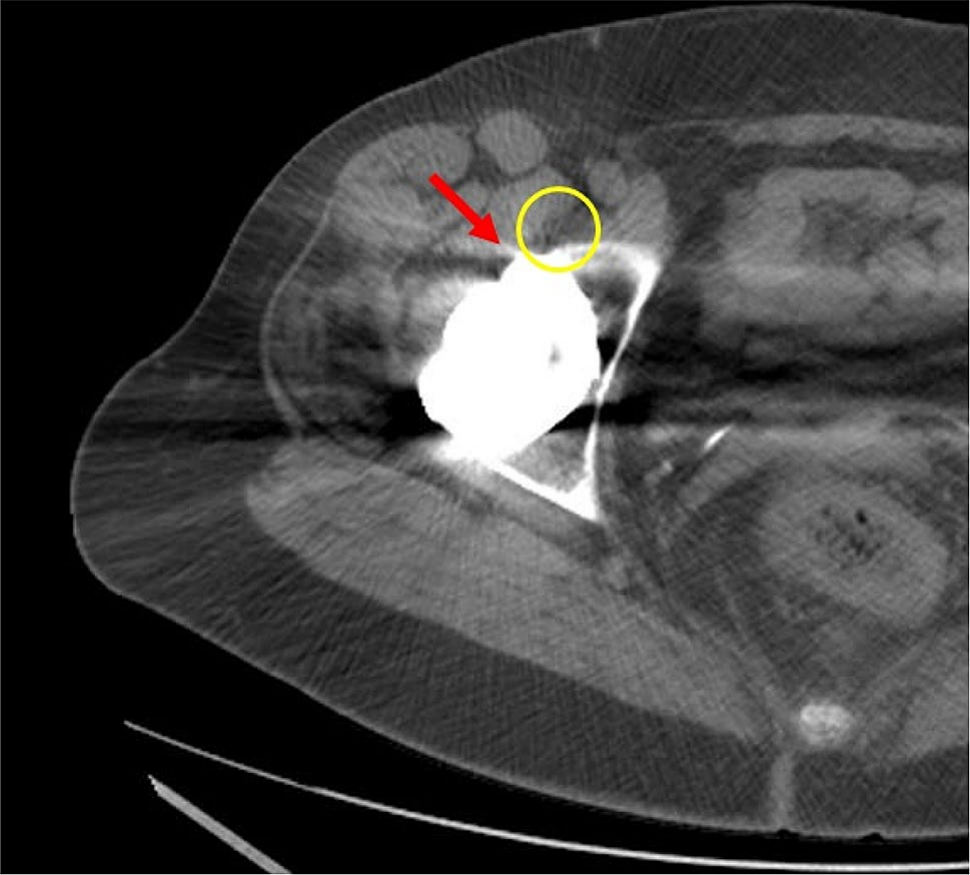
Axial CT scan of a right hip, red arrow: prominent acetabular cup; yellow circle: iliopsoas tendon irritated by acetabular cup prominence

Diagnosis was founded on clinical and imaging evidence that was based on AP-pelvic and cross-lateral radiographs and computed tomography (Fig. 1). In addition, all patients received a sonography-guided corticosteroid injection in the psoas tendon sheath to confirm iliopsoas impingement as the source of pain. The local infiltration test was considered positive if the patient reported pain relief after infiltration. None of the included patients showed signs of infection, implant loosening, or a prominence of the acetabular component of more than 8 mm. All patients had undergone conservative therapy for at least 6 months before surgery had to be indicated due to persistent pain. The primary clinical outcomes analyzed were the modified Harris Hip Score (mHHS) [30, 31], visual analogue scale (VAS) pain and University of California Los Angeles (UCLA) activity score [32], that were assessed preoperatively and at the time of follow-up. Informed consent was obtained from all individual participants included in the study. The Strengthening the Reporting of Observational Studies in Epidemiology (STROBE) checklist for cohort studies has been applied [33].

### Surgical technique

**Fig. 2:**
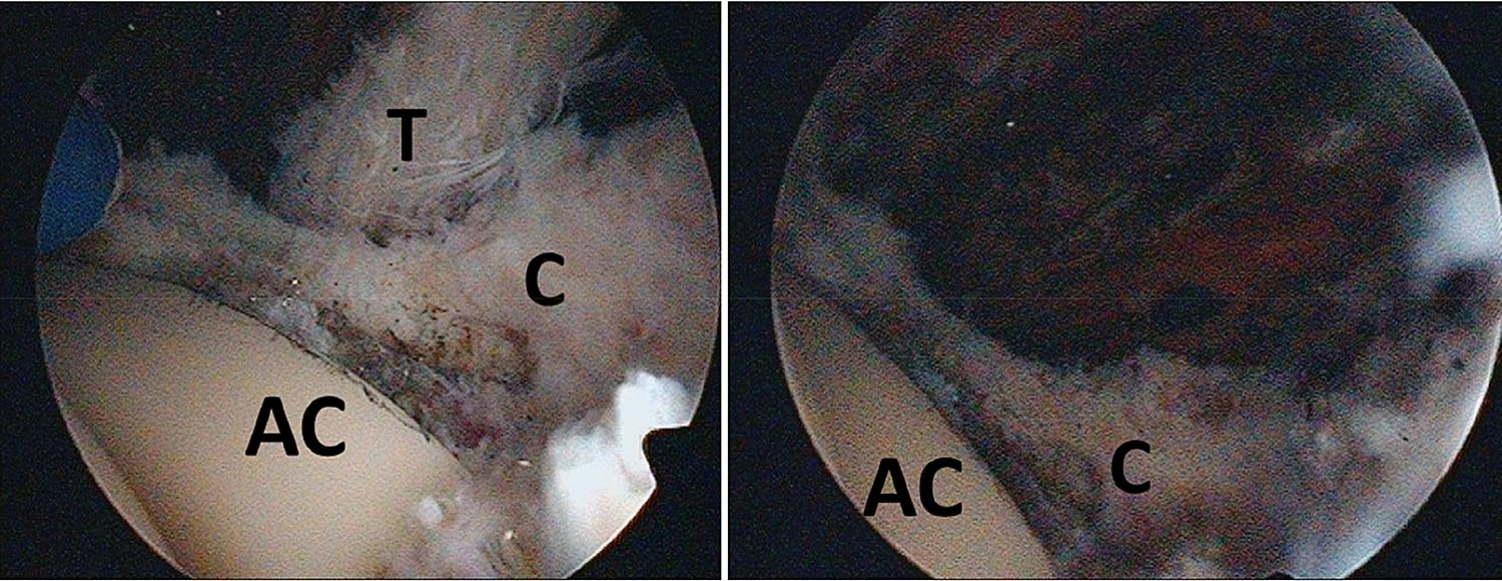
Left: arthroscopic view onto a left hip prosthesis, the iliopsoas tendon is irritated by the acetabular component; right: situation after arthroscopic tenotomy using a radiofrequency device. *T* tendon, *C* capsule, *AC* acetabular component

The surgical procedure is performed under general anesthesia in the supine position using a traction table and a perineal post. The hip is prepared and draped in the usual fashion. Two standard portals are used for each arthroscopy and established under the use of fluoroscopy (proximal anterior and midanterior portal). The 70° arthroscope is inserted through the proximal anterior portal and a radiofrequency device through the midanterior portal. First scar tissue is dissected until the head and socket are identified. Then a capsulotomy is performed at the iliopsoas notch. After identifying the iliopsoas tendon, it is released at the impingement zone using a radiofrequency device or a shaver (Fig. 2).

### Rehabilitation

For the first 2 weeks, hip flexion is pain-dependent permitted and the patient can be mobilized pain-adapted to crutches. Physical therapy starts on the first postoperative day. After 2 weeks, the crutches are discontinued and physical therapy should focus on strengthening the gluteus medius and core muscles, as well as progression of range of motion.

### Statistical analysis

Statistical analyses were performed using SPSS Statistics 24 (IBM; Armonk, NY). Continuous variables were compared using unpaired Student’s *t* test. Categorical variables were tested using the Fisher exact test. To determine whether there are preoperative patient characteristics that may have some influence on the outcome a Spearman’s correlation analysis was conducted. Variables used in the analysis were age, gender, BMI, smoking status, and symptom duration in month before surgery. A *p* value of less than 0.05 was considered to be statistically significant.

## Results

Between January 2007 and December 2016, a total of 25 patients received arthroscopic release of the iliopsoas tendon. Of these 25 patients, 2 had died in the meantime, 2 had moved and were lost to follow-up and 1 refused to participate. Data of 20 patients were available at mean follow-up of 7 ± 3.8 (2.6–11.7) years. Mean age at arthroscopy was 59 ± 27.7 (52–78) years. Mean BMI was 25.7 ± 5.5 (20.4–34.5) kg/m^2^. Mean interval between THA and arthroscopy was 6.3 ± 4.0 (1.7–15) years. The gender ratio was 1:1 (10 males, 10 females). All patients were treated with a cementless cup and a cementless stem. The laterality showed twelve right and eight left hips. The patients included had direct lateral (13 patients) or dorsal surgical approach (7 patients). None of the patients had an anterior approach. Radiological analysis showed correct cup inclination (40°–50°) in 16 patients (80%). In four patients (20%), the cup was found to be more vertical (> 50°). Two patients had an anteversion of the acetabular component of < 10°. The mean prominence of the acetabular component was 5.5 ± 1.8 (2–8) mm.

Before hip arthroscopy each patient reported pain with hip flexion, five patients complained of occasional snapping. The ability to flex was painfully limited in all patients; however, active flexion against resistance was possible without any weakness. Pain location was 95% inguinal, 40% thigh and 25% adductors.

At follow-up, there were no complications such as venous thrombosis, heterotopic ossifications, or further operations. 90% (18/20) of patients had a resolution of pain, 2 patients still reported some residual symptoms, but these were markedly less than preoperative. All five patients with occasional snapping did not complain of any further snapping phenomena. Modified Harris Hips Score and VAS pain showed significant improvements at follow-up (*p* = 0.0001). UCLA activity score showed an improvement but without statistical significance (*p* = 0.09) (Table 1).

**Table 1:** Clinical outcome: values are shown as mean ± SD (range)

Variable	Preoperative	Follow-up	Difference preop follow-up	*p* value
mHHS	31.2 ± 9.8 (17.6–47.3)	82 ± 9.8 (46.2–100)	50.8 ± 23.8 (26.4–69.3)	< 0.0001
Pain VAS	8.5 ± 1.2 (7–10)	2.5 ± 1.8 (0–6)	6.0 ± 1.2 (4–8)	< 0.0001
UCLA score	4 ± 2.7 (0–7)	6.5 ± 1.8 (3–9)	2.5 ± 2–0 (0–7)	0.09

*mHHS* modified Harris Hip Score, *pain VAS* nvisual analogue scale

The correlation analysis showed that BMI had a significant influence on the improvement achieved in mHHS and VAS pain: a higher BMI was significantly associated with a higher improvement in mHHS (*r* = 0.755, *p* = 0.046) and VAS pain (*r* = 0.835, *p* = 0.015). Age, gender, smoking status, and duration of symptoms had no influence on the results.

## Discussion

This study found good clinical results and significant improvements in mHHS and VAS pain in the mid-term after arthroscopic release of the iliopsoas tendon in the case of impingement due to THA. To the best of our knowledge, this study shows the longest published follow-up period with 7 years. Jerosch et al. [29] reported a follow-up of 6.5 years for 68 patients.

Iliopsoas impingement is a cause of persistent inguinal pain after THA. The primary therapy should be of a non-surgical nature including physiotherapy, anti-inflammatory medications, and injections. Studies have shown pain resolution through conservative therapy in up to 56% of the patients [5, 24]. However, some studies reported no improvement after conservative therapy or a lower rate of pain reduction compared to surgical treatment [10, 24].

If there is no improvement after conservative therapy, surgical treatment is recommended. Several options have been described in the literature: revision of the acetabular component or an open or endoscopic/arthroscopic release of the iliopsoas tendon. Few authors reported the results after acetabular component revision [10, 24, 34]. In these studies, the Harris Hip Score (HHS) could be significantly improved to values between 76 and 82 points [10, 24]. However, the authors reported a complication rate between 6.5 and 50% [10, 34].

In contrast to cup revision, minimally invasive procedures are also feasible by tenotomy of the iliopsoas tendon which can be conducted open or arthroscopically/endoscopically. An open tenotomy can be performed using a posterior approach as well as a lateral or anterior approach. Successful outcomes were described in 81–83% of the cases [10, 12, 36]. With the advancement of hip arthroscopy, arthroscopic/endoscopic techniques have also gained attention. Consequently, several publications report good results with pain alleviation in 80–92% after arthroscopic/endoscopic iliopsoas release [11, 22, 28, 37, 38]. However, most studies only report short-term results. Our study now shows that the good results can be sustained over the medium term. Table 2 shows an overview of the existing literature and the respective results after arthroscopic/endoscopic release of the iliopsoas tendon.

**Table 2:** Overview of existing literature after arthroscopic/endoscopic iliopsoas tenotomy

Study	Sample size (patients)	Mean age (years) (range)	% Female	F/U (years)	Mean time from prev. index surgery (years) (range)	Index arthroplasty	Pain relief (%)	PROM	PROM preop Mean (range)	PROM F/U Mean (range)	*p* value
Bajwa et al. [40]^a^	5	NR	NR	NR	NR	1 THA, 3 R, 1 PR	NR	NR	NR	NR	NR
Van Riet et al. [23]	9	51 (24–81)	NR	0.9	0.5	4 THA, 4 R	NR	HOOS	41 (11–53)	58 (32.5–100)	N.S.
Pattyn et al. [41]^a^	3	55.3 (53–57)	66	NSE	1.25	3 THA	NSE	NR	NR	NR	NR
Dallari et al. [42]	7	NSE	NSE	NSE	NSE	NSE	86	NSE	NSE	NSE	NSE
Lahner et al. [43]^a^	2	55.5 (61–60)	50	NSE	0.9	2 THA	100	HOS ADL	57.85	87.45	NR
Gédouin et al. [22]	10	58 (45–80)	50	1.7	NR	9 THA, 1 R	80	WOMAC	34 (24–46)	84 (60–95)	NR
Lindner et al. [44]	1	51	100	0.25	NR	1 THA (collared femoral stem)	100	NR	NR	NR	NR
Mei-Dan et al. [45]^a^	17	NSE	NSE	NSE	NSE	17 R	NSE	WOMAC	NSE	NSE	NR
Filanti et al. [37]^a^	7	54.9 (29–77)	NSE	NSE	0.9	6 THA, 1 R	NSE	HHS	46.4 (32–56)	83.3 (61–91)	NR
Jerosch et al. [11]	35	NR (58–82)	NR	3.6	NR	23 THA, 12 R	94	NR	NR	NR	NR
Jerosch et al. [29]	68	63 (45–77)	62	6.5	0.75	53 THA, 15 R	96	HHS	42.1 (33–55)	85.2 (63–95)	NR
Guicherd et al. [28]	64	56.3 (33–78)	63	0.67	3.36 (0.33–12)	64 THA	92	OHS	21.8	40	< 0.001
Williams et al. [26]	13	52.8 (29.1–82.7)	85		2.9 (0.4–10.1)	13 THA	62	NR	NR	NR	NR
Moreta et al. [38]	12	59.1 (40–72)	50	3.75 (2–8)	1.67 (0.67–2.17)	12 THA	91.7	mHHS	58.8 (37–76)	86.1 (59–98)	0.001

*F/U* follow-up, *NR* not recorded, *NSE* not specially evaluated, *N.S.* not significant, *THA* total hip arthroplasty, *R* resurfacing, *PR* partial resurfacing, *PRO* patient relates outcome measurement, *HOOS* Hip Injury and Osteoarthritis Outcome Score, *HOS ADL* Hip Outcome Score Activities of Daily Living, *WOMAC* Western Ontario and McMaster Universities Osteoarthritis Index, *HHS* Harris Hip Score, *OHS* Oxford Hip Score

^a^Results are part of a subgroup analysis

The tenotomy can be performed either at the level of the lesser trochanter or transcapsular, whereby an outside-in and inside-out method is described for the transcapsular technique [38]. An anatomical study has shown that the muscle volume is greater at the level of the joint space than at the level of the lesser trochanter [39], which is why we recommend a transcapsular tenotomy to preserve muscular tissue. In addition, the THA can be evaluated and possible scar tissue can be removed.

To help in deciding whether a cup revision or a psoas release should be performed, Chalmers et al. investigated the influence of the amount of the acetabular component prominence. In their study, a revision of the acetabular component showed a significantly higher pain resolution compared to iliopsoas tenotomy in cases with a acetabular component prominence of > 7 mm [24]. The mean acetabular component prominence in our study was 5.5 mm, so that we can recommend arthroscopic treatment in cases of cup prominence < 8 mm. With regard to a more prominent acetabular component, further studies should be performed to be able to develop a corresponding therapy recommendation.

Our study also investigated the influence of arthroscopic tenotomy on athletic activity. We were able to show a minimal improvement of the UCLA activity score. None of the above-mentioned studies examined the athletic ability based on the UCLA activity score.

Looking at the results of this study as well as the other publications mentioned above, results after an arthroscopic/endoscopic release provide improvement and good overall scores. However, scores in patients with THA not suffering from iliopsoas impingement are higher and show better overall outcomes. Usually the (modified) Harris Hip Scores are over 90 points [35]. One reason for this may be continued irritation of the anterior capsule and soft tissue structures caused by minimal prominence of the cup component. However, good results can be achieved with the endoscopic and arthroscopic technique with the advantage that it is less invasive compared to the open technique or even revision of the acetabular component. Another advantage of the endoscopic procedure is the very low complication rate compared to the more invasive acetabular component revision.

The current study has some limitations. First it is a retrospective study. The included group was small with 20 patients. However, most published studies reported smaller groups with 6–10 patients [10, 23, 24]. Jerosch et al. [29] reported the highest sample size with 68 patients in 2017. A further limitation is the missing control group, whereby in the present population no cup prominence of more than 8 mm was present and consequently, according to the Chalmers study, an release of the iliopsoas tendon should be sufficient [24].

One of the main strengths of our study is the long follow-up time of 7 years. To our knowledge, our study has the longest follow-up period. Another strength of the study is that all operations were performed by one surgeon (W.M.).

## Conclusion

Mechanical irritation and impingement of the iliopsoas tendon is a differential diagnosis to be considered in persistent groin pain after total hip arthroplasty. Patients show typical symptoms such as painful active flexion of the hip. The therapy of choice is initially conservative. However, with frustrating conservative therapy, good clinical results can be achieved with arthroscopic release and the pain level can be significantly reduced with a very low complication rate. The endoscopic release, therefore, represents a minimally invasive alternative to acetabular component revision.
