# Correction to: Results after arthroscopic treatment of iliopsoas impingement after total hip arthroplasty

**DOI:** 10.1007/s00402-021-03986-x

**Published:** 2021-06-22

**Authors:** A. Zimmerer, M. Hauschild, R. Nietschke, M. M. Schneider, G. Wassilew, C. Sobau, W. Miehlke

**Affiliations:** 1 ARCUS Sportklinik, Rastatterstr. 17-19, 75179 Pforzheim, Germany; 2 Department of Orthopedics and Orthopedic Surgery, University Medicine Greifswald, Ferdinand-Sauerbruch-Straße, 17475 Greifswald, Germany; 3 University of Witten/Herdecke, Alfred-Herrhausen-Straße 50, 58455 Witten, Germany

## Correction to: Archives of Orthopaedic and Trauma Surgery 10.1007/s00402-020-03623-z

The article Results after arthroscopic treatment of iliopsoas impingement after total hip arthroplasty, written by A. Zimmerer, M. Hauschild, R. Nietschke, M. M. Schneider, G. Wassilew, C. Sobau, W. Miehlke, was originally published electronically on the publisher’s internet portal on 12 October 2020 without open access. With the author(s)’ decision to opt for Open Choice the copyright of the article changed on 25 May 2021 to © The Author(s) 2021 and this article is licensed under a Creative Commons Attribution 4.0 International License, which permits use, sharing, adaptation, distribution and reproduction in any medium or format, as long as you give appropriate credit to the original author(s) and the source, provide a link to the Creative Commons licence, and indicate if changes were made. The images or other third party material in this article are included in the article’s Creative Commons licence, unless indicated otherwise in a credit line to the material. If material is not included in the article’s Creative Commons licence and your intended use is not permitted by statutory regulation or exceeds the permitted use, you will need to obtain permission directly from the copyright holder. To view a copy of this licence, visit http://creativecommons.org/licenses/by/4.0/.

The original article has been corrected.

